# Polymorphisms in the *SULF1 *gene are associated with early age of onset and survival of ovarian cancer

**DOI:** 10.1186/1756-9966-30-5

**Published:** 2011-01-07

**Authors:** Chan H Han, Yu-Jing Huang, Karen H Lu, Zhensheng Liu, Gordon B Mills, Qingyi Wei, Li-E Wang

**Affiliations:** 1 Department of Epidemiology, The University of Texas M. D. Anderson Cancer Center, Houston, TX 77030, USA; 2 Department of Gynecologic Oncology, The University of Texas M. D. Anderson Cancer Center, Houston, TX 77030, USA; 3 Department of Systems Biology, The University of Texas M. D. Anderson Cancer Center, Houston, TX 77030, USA

## Abstract

**Background:**

SULF1 (sulfatase 1) selectively removes the 6-O-sulphate group from heparan sulfate, changing the binding sites for extracellular growth factors. *SULF1 *expression has been reported to be decreased in various cancers, including ovarian cancer. We hypothesized that single nucleotide polymorphisms (SNPs) of *SULF1 *would impact clinicopathologic characteristics.

**Methods:**

We genotyped five common (minor allele frequency>0.05) regulatory SNPs with predicted functionalities (rs2623047 G>A, rs13264163 A>G, rs6990375 G>A, rs3802278 G>A, and rs3087714 C>T) in 168 patients with primary epithelial ovarian cancer, using the polymerase chain reaction-restriction fragment length polymorphism method.

**Results:**

We found that rs2623047 G>A was significantly associated with an early age of onset of ovarian cancer in the G allele dose-response manner (*P *= 0.027; *P_trend _*= 0.007) and that rs2623047 GG/GA genotypes were associated with longer progression-free survival; rs6990375 G>A was also associated with the early age of onset in the A allele dose-response manner (*P *= 0.013; *P_trend_*= 0.009). The significant differences in age of disease onset persisted among carriers of haplotypes of rs2623047 and rs6990375 (*P *= 0.014; *P_trend _*= 0.004). In luciferase reporter gene assays, rs2623047 G allele showed a slightly higher promoter activity than the A allele in the SKOV3 tumorigenic cell line.

**Conclusions:**

These findings suggest that genetic variations in *SULF1 *may play a role in ovarian cancer onset and prognosis. Further studies with large sample sizes and of the mechanistic relevance of *SULF1 *SNPs are warranted.

## Background

SULF1 is a newly identified human sulfatase with aryl-sulfatase activities, which can influence the sulfation status and biological function of heparan sulfate proteoglycans (HSPGs) [1]. This heparan sulfate 6-O-endosulfatase selectively removes 6-O-sulphate group and alters the binding sites of signaling molecules [2]. HSPGs are protein-conjugated forms of heparin sulfate glycosaminoglycans (HSGAGs) *in vivo *and major constituents of the extracellular matrix (ECM). HSGAGs in the ECM interact with many signaling molecules, regulate their biological activities, and express profound effects on cell growth kinetics and metastasis of tumor cells [3,4]. By interacting with numerous mediators including growth factors, cytokines, chemokines, and adhesion molecules, HSGAGs are involved in a wide array of biological processes, such as homeostasis, anticoagulation, angiogenesis, embryogenesis, as well as in oncogenic transformation of normal cells to tumor cells [5-10].

The correlation between *SULF1 *and cancer risk has mainly been studied in terms of gene expression. *SULF1 *expression is decreased in multiple malignant lineages, and its re-expression is known to be associated with decreased signaling of heparin-binding growth factors, cell proliferation, and the invasiveness of cancer cells [11-14]. In ovarian cancer, decreased expression of *SULF1 *and its correlation with decreased sensitivity to cisplatin (a standard chemotherapeutic agent) were also reported [12,15].

Loss of heterozygosity or hypermethylation of the promoter region has been suggested as potential mechanisms for *SULF1 *down-regulation in ovarian cancer [14]. Besides, genetic variation has been implicated in altered gene expression, especially those regulatory polymorphisms that are located in promoter regions [16,17]. However, genetic variation in *SULF1 *has not been explored in ovarian cancer. In this study, we genotyped five common (i.e. minor allele frequency>0.05) single nucleotide polymorphisms (SNPs) with predicted functionalities (rs2623047 G>A, rs13264163 A>G, rs6990375 G>A, rs3802278 G>A, and rs3087714 C>T ) to evaluate associations between these potentially functional *SULF1 *SNPs and clinical outcomes in 168 ovarian cancer patients whose DNA and clinic variables were available, and investigated whether the promoter activity of rs2623047 A>G may be underlying the functional significance.

## Methods

### Study Population

The study population and data collection were described previously [18]. Briefly, the 168 patients were registered at The University of Texas M. D. Anderson Cancer Center between 2000 and 2007 and diagnosed with histopathologically confirmed primary epithelial ovarian cancer. Patients had been treated with chemotherapy, a combination of platinum (carboplatin, cisplatin) and taxanes (taxol, docetaxel) following optimal debulking or cyto-reductive surgery. Available demographic characteristics included age at diagnosis and race, and clinicopathologic characteristics including tumor stage, cell type and grade, optimality of the primary debulking operation, chemotherapy regimen, number of chemotherapies, disease recurrence, and response of tumors to chemotherapy. The optimal debulking or cyto-reductive surgery is defined as the largest residual tumor nodule measuring 1 cm or less, according to the Gynecologic Oncology Group [19]. The response evaluation criteria in solid tumors (RECIST) [20] were used to define the response of tumors to treatment.

Overall survival (OS) and progression-free survival (PFS) were calculated as the date of disease diagnosis to the date of death or last contact or the date of recurrence or progression, accordingly. Disease recurrence was defined as the reappearance of any lesion that had previously disappeared or the appearance of a new lesion that was histopathologically confirmed by a biopsy. Information about the date of last contact and status of patients at the last contact was obtained from the M. D. Anderson Tumor Registry and Social Security Death Index, when this information was missing from the medical records. This study was approved by the M.D. Anderson Institutional Review Board.

### SNP Selection and Genotyping

Using *SULF1 *gene position from International HapMap project http://hapmap.ncbi.nlm.nih.gov/cgi-perl/gbrowse/hapmap28_B36/#search with the extension of 2 kb at both sides to cover near gene regions (chr8:70539427..70737701), we found that five of 355 SNPs were common in HapMap Caucasian population with one of following predicted functionalities at the SNP Function Prediction website http://snpinfo.niehs.nih.gov/snpfunc.htm: (1) affecting transcription factor binding sites (TFBS) activity in the putative promoter region, (2) affecting splicing activity, or (3) affecting the microRNA binding sites activity. Therefore, we genotyped all of these five SNPs: rs2623047 G>A, rs13264163 A>G, rs6990375 G>A, rs3802278 G>A, and rs3087714 C>T.

**Figure 1:**
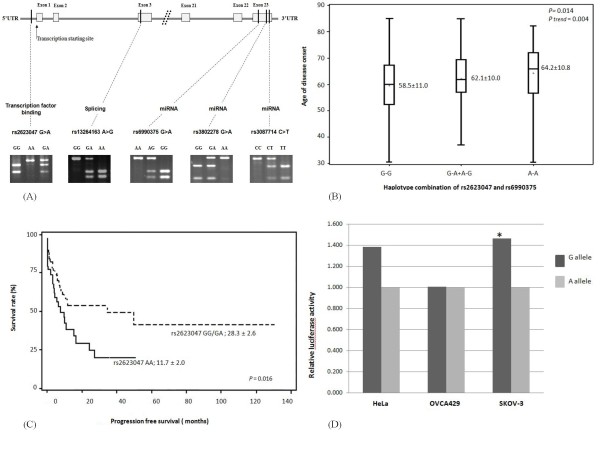
***SULF1 *SNP information, effects on age of disease onset, survival, and promoter activity**. (A) The gene structure, SNP location, predicted functionality of SNPs, and electrophoresis gel pictures; (B) Haplotype combination of rs2623047 and rs6990375 and age of disease onset; G-G: rs2623047G-rs6990375G; G-A/A-G: rs2623047G-rs6990375A and rs2623047A-rs6990375G; A-A: rs2623047A-rs6990375A; (C) Progression-free survival; rs2623047 AA vs. rs2623047 GG/GA; (D) HeLa, OVCA429, and SKOV-3 cell lines were co-transfected with the rs2623047 G, or rs2623047 A constructor plasmid and Renilla-TK plasmids. The relative luciferase activity was assessed with the *Renilla luciferase *activity. Each experiment was performed in triplicate. * *P *< 0.05.

The genotyping was performed by the polymerase chain reaction-restriction fragment length polymorphism method (PCR-RFLP) using genomic DNA. Table 1 shows the primers and PCR information for each SNP. The PCR conditions consisted of an initial melting step of 95°C for 5 min, followed by 35 cycles of denaturation (95 °C for 30 seconds), annealing (52 - 55 °C for 45 sec according to SNPs), and extension (72°C for 1 min), and a final extension step of 72°C for 10 min. The digested products were checked on a 3% MetaPhor agarose gel containing ethidium bromide. The gene structure, SNP location, predicted functionality of SNPs, and electrophoresis gel pictures are shown in Figure 1A. The genotypes were double-checked by two people for quality control, and any uncertain results were repeated to reach a 100% concordance. Genotyping of 10% of samples were randomly performed twice, and no discrepancy was observed.

**Table 1:** Primers and PCR conditions for genotyping the five SNPs

rs number		Primers	Annealing Temperature (°C)	PCR products (bp)	Enzyme	Digested PCR products (bp)
rs2623047	FP	5'-TGT GGC AAA CAG TGA AGA GC-3	52	245	*BstNI*	GG:159/86
G>A	RP	5'-CAG CAA GAC GTT TTC CCT TC-3'				GA:245/159/86
						AA:245
rs13264163	FP	5'-TGG CAA TTT TGC TCT TTT CC-3'	55	181	*NspI*	AA:100/81
A>G	RP	5'-TGA CAT AGA GTG CCC AGG TG-3				GA:181/100/81
						GG:181 G
rs6990375	FP	5'-CCG CAG AAC ACC GAA GTA AT-3'	55	227	*HhaI*	GG:128/99
G>A	RP	5'-CCA GGG TAG CTT GGA ATG TT-3				GA:227/128/99
						AA:227
rs3802278	FP	5'-CTG GAA ACC GAT TTC AGT GG-3'	55	227	*Cac8I*	GG:151/76
G>A	RP	5'-CCC GCT ATG CTG GAA TTA CT-3				GA:227/151/76
						AA:227
rs3087714	FP	5'- TTC CTG AAG CCA GAA TTG TTC-3'	55	150	*CviQI*	CC:150
C>T	RP	5'- TAT CAT CGG TGG GAT GAC AG-3'				CT:150/101/49
						TT:101/49

### Construction of Reporter Plasmids

Reporter constructs were prepared for rs2623047 G>A by amplifying 1803 bp of the *SULF1 *promoter region (from -1784 to +18 relative to the transcription start site) with either rs2623047 G or A allele by using a pair of primers 5'-AAGAGCTCTTGGGAATGCCTCATAGACAG-3' (forward) and 5'-AAGCTAGCGGTCTGAGAACTCCCAGTCAA-3' (reverse). *SacI *and *NheI *restriction enzymes (New England BioLabs, Beverly, MA) were used to cleave the amplicons, and the pGL4 vector (Promega, Madison, WI) and T4 DNA ligase (New England BioLabs) were used for ligation.

### Transient Transfection and Luciferase Reporter Gene Assay

The ovarian cancer cell lines OVCA429 and SKOV-3 were cultured in 1x McCoy's 5A modified medium and minimum essential medium, and the human cervical cancer cell line HeLa was cultured in Dulbecco's modified Eagle's medium, supplemented with 10% fetal bovine serum (Sigma-Aldrich, MO) at 37°C with 5% CO_2_. The cultured cells were transiently transfected with 1.0 μg of rs2623047 G or rs2623047 A reporter constructs, using the FuGENE HD kit (Roche Applied Science, IN). The p-TK *renilla *luciferase (pRL-TK) (Promega) construct was co-transfected as an internal control to evaluate experimental variation, such as transfection efficiency and cell viability. The luciferase activities were quantified by a Dual-Luciferase Reporter Assay System (Promega), and the relative luciferase activity was calculated as the ratio of firefly to *renilla *luciferase activity, according to the manufacturer's instructions. Each experiment was repeated three times.

### Statistical Analysis

Statistical analysis was performed using the Chi-square test or analysis of variance (ANOVA) analysis for categorical variables and continuous variables, respectively. The Proc Allele procedure in the SAS/Genetics program (SAS Institute Inc., Cary, NC) was used to calculate linkage disequilibrium (LD). The Kaplan-Meier method and the log-rank test were used to estimate PFS and OS. The Cox proportional hazards regression model was used to analyze individual prognostic factors. All statistical tests were two-sided, a *P *value of 0.05 was considered statistically significant, and all analyses were performed using the Statistical Analysis System/Genetics software (SAS version 9.13; SAS Institute Inc.)

## Results

Demographic and clinicopathologic characteristics of the study population have been described elsewhere [18]. Since there are significant racial differences in allele distributions of some *SULF1 *SNPs and the majority of the patients with available DNA samples were non-Hispanic whites (136/168, 80.9%), we included non-Hispanic whites only in further analysis. As shown in Table 2 of clinicopathologic characteristics in this study, the mean age of disease onset and standard deviation (SD) was 61.8 ± 10.7 years, and 12.5% were younger than 50 years. Among the 136 white patients, 91.9% had an advanced disease with 102 patients (75.6%) diagnosed at stage III and 22 patients (16.3%) diagnosed at stage IV. Most patients had high grade (127, 95.5%) and serous cell type (109, 80.2%), and 85 patients (62.5%) had obtained optimal debulking during primary surgery.

**Table 2:** Demographic and clinicopathologic characteristics in non-Hispanic white ovarian cancer patients

Characteristics	Number of patients	%
Age at Diagnosis (years)	136	
<50	17	12.5
50 - 70	86	63.2
>70	33	24.3
Surgical stage ^a^	135	
I	5	3.7
II	6	4.4
III	102	75.6
IV	22	16.3
Tumor Grade ^a^	133	
1	6	4.5
3	127	95.5
Histology	136	
Serous	109	80.2
Mucinous	2	1.5
Endometrioid	2	1.5
Clear cell	1	0.7
Brenner	3	2.2
Mixed	19	14.0

^a ^Missing patient information: 1 for surgical stage; 3 for tumor grade

Table 3 shows genotype distribution of the five SNPs. The LD analysis showed disequilibrium coefficient D' = 0.965 and Correlation coefficient *r*^2 ^= 0.872 for rs6990375 G>A and rs3802278 G>A; D' = 0.981 and *r*^2 ^= 0.678 for rs6990375 G>A and rs3087714 C>T; D' = 1.000 and *r*^2 ^= 0.919 for rs3802278 G>A and rs3087714 C>T, but other pairs showed lower D' and *r*^2 ^values, suggesting that rs6990375 G>A can capture the majority of rs3802278 G>A and rs3087714 C>T changes in the 5' UTR. When we stratified the age of disease onset by these genotypes, we found that all five SNPs were more or less associated with age of onset of ovarian cancer. For example, the rs2623047 G>A showed an association with age of disease onset (Table 3); the patients with the AA genotype had a mean age of onset of 65.0 ± 9.9 years; and those with the AG genotype had 61.2 ± 10.8 years, while those with the rs2623047 GG showed 56.8 ± 10.7 year age of onset (*P *= 0.027 for the ANOVA test). The trend test showed a *P *value of 0.007 for a decreasing age with the G allele in a dose-dependent manner (Table 3). The rs13264163 AG heterozygotes also showed the youngest age of onset among all genotypes of rs13264163A>G (*P *= 0.016) (Table 3). We also found that the early age of disease onset was associated with the G allele of rs6990375 G>A [rs6990375 GG: 60.0 ± 10.7 years; rs6990375 GA: 61.8 ± 10.6 years; rs6990375 AA: 69.1 ± 9.0 years (*P *= 0.013)] (Table 3). As we noticed in the LD analysis, rs6990375 G>A had a *r*^2^> 0.8 with rs3802278 G>A and rs3087714 C>T; therefore, we also observed the significant trends in differences of age of disease onset among genotypes of rs3802278 G>A and rs3087714 C>T (*P_trend _*= 0.021 and 0.041, respectively), even though the differences were not significant in ANOVA tests (*P *= 0.069 and 0.119).

**Table 3:** *SULF1*Genotype distribution and age of disease onset

Genotypes	Number of patients (%)	Age at diagnosis (years, mean ±SD) ^b^	*P-*value
rs2623047 G>A ^a ^			0.027
GG	16 (11.9)	56.8 ± 10.7	
GA	80 (59.3)	61.2 ± 10.8	
AA	39 (28.9)	65.0 ± 9.9	
G allele frequency	112 (41.5)		*P_trend _*^c ^= 0.007
A allele frequency	158 (58.5)		
rs13264163 A>G			0.016
AA	70 (51.4)	63.7 ± 10.5	
AG	53 (39.0)	58.6 ± 10.5	
GG	13 (9.6)	64.9 ± 10.6	
A allele frequency	193 (71.0)		*P_trend _*^c ^= 0.266
G allele frequency	79 (29.0)		
rs6990375 G>A			0.013
GG	58 (42.7)	60.0 ± 10.7	
GA	63 (46.3)	61.8 ± 10.6	
AA	15 (11.0)	69.1 ± 9.0	
G allele frequency	179 (65.8)		*P_trend _*^c ^= 0.009
A allele frequency	93 (34.2)		
rs3802278 G>A			0.069
GG	59 (43.4)	59.7 ± 11.4	
GA	65 (47.8)	62.8 ± 10.0	
AA	12 (8.8)	66.7 ± 9.5	
G allele frequency	183 (67.3)		*P_trend _*^c ^= 0.021
A allele frequency	89 (32.7)		
rs3087714 C>T			0.119
CC	63 (46.3)	60.1 ± 11.3	
CT	62 (45.6)	62.7 ± 10.1	
TT	11 (8.1)	66.6 ± 10.0	
C allele frequency	188 (69.1)		*P_trend _*^c ^= 0.041
T allele frequency	84 (30.9)		

^a ^One sample failed in this genotype

^b ^One-way ANOVA (Analysis of variance) for age differences among 3 genotypes for each SNP

^c ^*P *values for the trend test of age at diagnosis among 3 genotypes for each SNP from a general

linear model

We further evaluated the combined allele effect on age of disease onset. Because rs2623047 G>A and rs6990375 G>A showed significant differences among genotypes and significant trends, and rs6990375 G>A is in LD with rs3802278 G>A and rs3087714 C>T, we only included those two SNPs in the haplotype analysis. The significant differences in age of disease onset remained among carriers of the haplotype of rs2623047G and rs6990375G as compared with other haplotypes (*P *= 0.014; *P_trend _*= 0.004) as shown in Figure 1B. In further analysis, we also found that rs2623047 A>G was associated with PFS. Patients with the G allele (i.e., the GG/GA genotypes) showed a longer PFS than patients with the AA genotype (28.3 ± 2.6 months vs. 11.7 ± 2.0 months; *P *= 0.016) (Figure 1C), whereas this association with PFS was not observed for other *SULF1 *SNPs.

Since rs2623047 is located in the putative promoter region of *SULF1*, we further tested its effect on the promoter activity. We constructed luciferase reporter plasmids with either rs2623047 G allele or rs2623047 A allele and transiently transfected them into three cancer cell lines, OVCA429, SKOV-3, and HeLa. We found that the *SULF1 *promoter containing rs2623047 G exhibited an increased luciferase activity, compared with the rs2623047 A in SKOV-3 and HeLa cell lines, but only SKOV-3 ovarian cancer cell lines showed a statistically significant difference (*P *= 0.028), whereas HeLa cells showed a marginal difference with a *P *value of 0.058 (Figure 1D). Intriguingly, it is known that OVCA 429 forms tumor slowly and less aggressively in nude mice [21,22], whereas SKOV-3 is highly tumorigenic [23], potentially relating to the differences in the promoter activity in the two lines.

## Discussion

SULF1 is a recently identified heparin-degrading endosulfatase, which catalyzes the 6-O desulfation of HSPGs, co-receptors for heparin-binding growth factors and cytokine signaling pathways [12-14,24-27]. Moreover, SULF1 has been linked with a tumor suppression function and its expression was ubiquitous but reportedly downregulated in most of cancer cell lines [28]. The mRNA expression of *SULF1 *has been reported to inhibit tumor growth and angiogenesis in breast cancer cell lines [29] and also altered cisplatin-treatment response in ovarian cancer [15].

In this study, we genotyped five putatively functional common *SULF1 *SNPs to investigate associations between these genetic variants and clinical outcomes in ovarian cancer patients. We found that all five SNPs were more or less associated with age of onset of ovarian cancer, especially rs2623047 G>A and rs6990375 G>A. We also found that rs2623047 G allele was associated with a longer PFS in the ovarian cancer patients, suggesting that carriers of the rs2623047 G allele may be more responsive to treatment. Our luciferase reporter gene assay of rs2623047 G>A further showed that the G allele exhibited slightly higher promoter activity in SKOV-3 and HeLa cancer cell lines, which is consistent with one published study in which ovarian cancer patients with higher expression of *SULF1 *were more sensitive to platinum chemotherapy compared to others with lower *SULF1 *expression [15], suggesting that the G allele had a tumor suppression effect. However, the biological relevance for an association between rs2623047 G allele and early onset of ovarian cancer remains unclear. It has been reported that multiple genetic or epigenetic changes are involved in signaling of certain growth factors leading to tumorigenesis [30-33], which may be potentially related to the SNP effects on the development of cancer. Although several studies reported that *SULF1 *expression was downregulated in different types of cancer [11-14], *SULF1 *was upregulated in gastric and pancreatic cancers [24,34]. A recent study also showed that *SULF1 *mRNA and protein expression were increased in the aging articular cartilage [35]. Therefore, our results call for additional replication studies with larger sample sizes and studies on possible mechanistic studies underlying the observed associations.

In the United States, epithelial cancer of the ovary is the fifth most common cause of death related to malignant conditions among women and the most leading cause of death from gynecologic malignancies [36]. Despite the fact that it is highly curable if diagnosed early, due to lack of symptoms in early stages of the disease, the majority of patients had presented with advanced diseases and subsequently had a worse prognosis. Unlike other cancers, there are no currently accepted standard screening tests to detect ovarian cancer at an early stage. More knowledge about ovarian cancer clinical characteristics will help develop more effective approaches to the disease. Hopefully in the future, our findings of the age difference by genetic variants could be a part of the efforts. However, our study had some limitations because of its small sample size. Additional studies with larger sample sizes with mechanistic studies to understand biological relevance of *SULF1 *SNPs in the development of ovarian cancer are needed to validate the role of *SULF1 *SNPs in age of disease onset and prognosis of ovarian cancer.

## Competing interests

The authors declare that they have no competing interests.

## Authors' contributions

CH participated in the study design and conducted the laboratory experiments, performed the statistical analysis, prepared figures, and tables and drafted the manuscript. YH performed the luciferase assay experiment in cell lines and participated the analysis and manuscript preparation. KHL provided patients' samples and clinical information. ZL advised on designing primers and helped laboratory experiments. GBM supported the study, provided information on the study design and edited the manuscript. QW advised on study design, and revised the manuscript preparation, and supported the study. L-EW participated in the study design, oversaw the entirety of the project and assisted in the analysis and the manuscript preparation. All authors read and approved the manuscript.
